# Dosimetric and clinical advantages of deep inspiration breath-hold (DIBH) during radiotherapy of breast cancer

**DOI:** 10.1186/1756-9966-32-88

**Published:** 2013-11-07

**Authors:** Vicente Bruzzaniti, Armando Abate, Paola Pinnarò, Marco D’Andrea, Erminia Infusino, Valeria Landoni, Antonella Soriani, Carolina Giordano, Anna Maria Ferraro, Lidia Strigari

**Affiliations:** 1 Laboratory of Medical Physics and Expert System, Regina Elena Cancer Institute, Via E. Chianesi 53, 00144 Rome, Italy; 2 Radiotherapy Department, Istituto Neurotraumatologico Italiano, Grottaferrata, Italy; 3 Radiotherapy Department, Regina Elena Cancer Institute, Rome, Italy; 4 Radiotherapy Department, Campus Bio-Medico University, Rome, Italy

**Keywords:** Deep inspiration breath-hold gating radiotherapy, Breast cancer radiotherapy, NTCP, TCP

## Abstract

**Background:**

To investigate the potential dosimetric and clinical benefits of Deep Inspiration Breath-Hold (DIBH) technique during radiotherapy of breast cancer compared with Free Breathing (FB).

**Methods:**

Eight left-sided breast cancer patients underwent a supervised breath hold during treatment. For each patient, two CT scans were acquired with and without breath hold, and virtual simulation was performed for conventional tangential fields, utilizing 6 or 15 MV photon fields. The resulting dose–volume histograms were calculated, and the volumes of heart/lung irradiated to given doses were assessed. The left anterior descending coronary artery (LAD) mean and maximum doses were calculated, together with tumour control probability (TCP) and normal tissue complication probabilities (NTCP) for lung and heart.

**Results:**

For all patients a reduction of at least 16% in lung mean dose and at least 20% in irradiated pulmonary volumes was observed when DIBH was applied. Heart and LAD maximum doses were decreased by more than 78% with DIBH. The NTCP values for pneumonitis and long term cardiac mortality were also reduced by about 11% with DIBH. The NTCP values for pericarditis were zero for both DIBH and FB.

**Conclusion:**

Delivering radiation in DIBH conditions the dose to the surrounding normal structures could be reduced, in particular heart, LAD and lung, due to increased distance between target and heart, and to reduced lung density.

## Background

With advances in mammography, breast cancer is being detected at an earlier stage and is therefore more curable [1]. The management of early breast cancer with conservative surgery and adjuvant whole radiotherapy is now a widely established alternative to mastectomy, which has long been the only accepted form of treatment [2]. Whole breast radiotherapy classically utilizes tangential fiels to encompass the entire breast volume and (tipically) wedge compensation are also used to ensure (a more oppure the better) homogeneous dose distribution. However, recent studies have shown that intrafraction target motion can decrease dose homogeneity [3-7] which is believed to be one of the main contributing factors to poor cosmesis and possibly to decreased tumor control [8].

The main cause of radiation underdosage in breast cancer patients can be attributed to the target motion due to respiration [2]. Breathing adapted radiotherapy of breast cancer seems to provide reduced radiation doses to Organs At Risk (OARs) without compromising Clinical Target Volume (CTV) coverage. Irradiation techniques have been developed to reduce the effects of motion, which can result in better dose homogeneity [2]. These techniques implies that the radiation beam is turned on only during a pre-specified phase or amplitude of the respiratory cycle, thus modifying target position and lung density within the field aperture. Several studies have reported that an appreciable reduction in cardiac volume within tangential radiation portals for left-sided breast cancer can be achieved by deep inspiration, either by a simple technique of non-monitored [9,10] or monitored [11] voluntary breath-hold, or by a complex technique of spirometrically monitored and forced breath-hold [12,13]. Additionally, they have also reported on pulmonary tissue sparing for both left- and right-sided cancers [11,13].

However, problems with breath-hold level reproducibility and verification, as well as with patient cooperation may limit the feasibility of this approach. Thus, the optimal parameters for the use of breathing control for breast cancer have not been established yet.

Korreman et al. [14] have investigated the possibility of decreasing chest wall excursion during breath-hold by audio-visually coaching the patient to a reproducible breath-hold level. The use of coaching appears to have the advantage of minimizing inter-session variability, and Kini et al. [15] have shown that such procedures may well allow a reduction of margins, implying even better normal tissue sparing.

A study by Stranzi and Zurl [16] demonstrates that during Deep Inspiration Breath-Hold (DIBH) technique, the left-sided breast and heart were separated during radiation treatment, thus excluding substantial heart volumes from the high-dose area.

The main objective of this study is to evaluate radiobiological, dosimetric effects and benefits of the DIBH technique during whole breast radiotherapy.

## Methods

Eight patients with left-early breast cancer who underwent conservative surgery and with a prescription of whole breast adjuvant radiotherapy were considered in this study. Patient eligibility criteria were: ≥ 18 years of age; not oxygen dependent; did not experience pain while in the supine position. Patient age ranged from 39 to 70 years (mean 51 years).

### Training

The training session established the patient’s inspiration level for treatment and breath-hold duration. A reflective marker (RPM Box) was placed on the patient’s abdominal surface, midway between the xyphoid process and the umbilicus to monitor the respiratory motion. The patients were asked to breathe freely and then inhale and hold their breath at a comfortable level, just below their maximum inspiration capacity, for at least 15 seconds. This cycle was to be repeated two or three times in succession. The respiratory signal was recorded with the Varian RPM™ system. Once a comfortable deep inspiration level was found, a lower and an upper thresholds were placed on the respiratory signal to define the gating window. The training was carried out by providing the patient with electronic eyeglasses (video coaching) which allowed visualization of a coloured band, representing the gating window, and a movable bar that followed the patient’s abdomen/chest movement, thus ensuring the reproducibility of deep inspiration amplitude. The width of the gating window was chosen such that the allowed amplitude of the residual RPM box motion was 0.5 cm. Under these conditions, a CT scan was performed for treatment planning. The patient had to be able to understand these instructions, be capable of performing a reproducible breath-hold, and be able to maintain it for at least 15 seconds. The training session required about 30 minutes.

### CT investigations

A CT Scanner Lightspeed 16 slices (GE) was used. The patients were placed in the treatment position supine with their arms raised above their head, the sternum in horizontal position and their shoulders, elbows and back immobilised with a wingboard. Orthogonal room lasers were used to place skin markers to verify that no shift occurred between scans. Finally, the RPM box was placed between the xyphoid process and the umbilicus, i.e. in proximity to the target breast, but outside the area to be covered by the radiation treatment fields. Two spiral scans were acquired, each covering the area from the mid-neck to the upper abdomen. The scanning parameters were: 120 kVp, mA range = 30–150 mA, 0.8 s/rotation, beam collimation = 20 mm, distance between two successive slices = 2.5 mm, image matrix = 512×512 pixels, field of view (FOV) = 50 cm. The first scan for conventional treatment planning (reference scan) was acquired during Free Breathing (FB). The second scan, acquired during DIBH, was manually started immediately after the inspiratory plateau was reached, as visually confirmed by the respiration monitoring. Both the antero-posterior FB motion amplitude during FB, and the maximum DIBH amplitude, of the RPM box, as detected by the RPM™ software, were registered for each patient. Whenever the CT scan acquisition was longer than the patient’s breath-hold time the scan was broken into two segments This happened for only one patient. The total time for the two CT acquistions was less than 15 minutes.

### Treatment planning

3D planning and dose computations were performed using the Anisotropic Analytical Algorithm (AAA) in the Eclipse treatment planning system (Varian Medical Systems, Palo Alto, USA). The planning CT scans consisted of 2.5 mm spaced slices of the whole chest, acquired during DIBH and FB. Structures such as body (external contour), Planning Target Volume (PTV), ipsilateral lung (IL), heart, anterior descending coronary artery (LAD) were delineated on both FB and DIBH reconstructed 3D-CT datasets.

Treatment plans were created using both CT data sets according to standard protocols. Two conventional 6 MV tangential opposed photons fields were generally used. For some patients a mixture of 6 and 15 MV photons fields were needed to improve target coverage. The fields were shaped with 120 leafs multileaf collimators, and wedges were used when appropriate for dose homogenization.

The two fractionation schedules currently in use in our Institute [17] were adopted. The first was a conventional treatment at 2 Gy daily fraction with a total dose of 50 Gy; the second was an hypofractionated treatment with a 3.4 Gy daily fraction up to 34 Gy total dose.

The plans were normalized to the target mean dose for the two breathing conditions (FB, DIBH). All targets were treated following internal criteria on dose homogeneity: 90% to 107% of the prescription dose. For each patient the Dose Volume Histograms (DVHs) of PTV, heart, IL and LAD were registered. From these data the mean and maximum doses of the IL, heart and LAD were extracted. In addition the percentage volume of the heart receiving more than 20 Gy and more than 40 Gy (V_20_(%) and V_40_(%)) and the percentage volume of the IL receiving more than 10 Gy and more than 20 Gy (V_10_(%) and V_20_(%)) were recorded. The central lung distance (CLD) [18], the absolute lung volume (ALV), i.e. the volume of the ipsilateral lung, the Irradiated Lung Volume (ILV), defined as the ipsilateral lung volume within the 50% isodose, the normalized irradiated lung volume (NILV) which is the ratio of ILV over ALV and the minimum distance between the heart and the target volume were measured on all the CT datasets.

### TCP and NTCP

Assuming that cell survival in a tumor follows a binomial statistic, the requirement of total eradication of all clonogenic cells yields the Poisson formula for Tumor Control Probability (TCP):

(1) $$\mathrm{TCP}=e^{-N^{*}\cdot \mathrm{SF}}$$

where N^*^ is the initial number of clonogenic tumor cells.

The Lyman-Kutcher-Burman (LKB) probit model [19] was used for calculating Normal Tissue Compliation Probability (NTCP). According to this model, if a fraction *v* of an organ is uniformly irradiated at dose D, the NTCP is given by the formula

(2) $$\text{NTCP}=\frac{1}{\sqrt{2\pi }}\int_{-\infty }^{t}\text{exp}\left(-\frac{t^{2}}{2}\right)\;\mathrm{dt}$$

where *t* is defined as,

(3) $$t=\frac{D\;-\;TD_{50}\left(v\right)}{m\;\cdot \;TD_{50}\left(v\right)}$$

and,

(4) $$TD_{50}\left(v\right)=TD_{50}\left(1\right)\;\cdot \;v^{-n}$$

The parameters n and m determine the volume dependence of NTCP and the slope of the curve NTCP vs. Dose, respectively. TD_50_(1) is the dose that leads to a 50% complication probability when it is delivered uniformly to the whole organ [19]. To estimate TD_50_(1) only standard fractionations of 1.8–2 Gy per day, 5 days per week, were considered [19]. As the irradiation of the organs at risk is almost never uniform, the effective volume method [19] is used as a histogram-reduction scheme for non-uniform organ irradiation:

(5) $$v_{\text{eff}}={\sum_{1}^{N}v_{1}\;\cdot \;\left(\frac{D_{1}}{D_{\text{max}}}\right)}^{1/n}$$

where D_*i*_ is the dose delivered to the volume fraction *v*_*i*_, and N is the number of bins of the differential DVH. By Eq. (4), an inhomogeneous dose distribution is converted to an equivalent uniform irradiation of a fraction *v*_*eff*_ of the organ at the maximum dose *D*_*max*_.

TCP and NTCP were calculated using the isoBED software [20] which applies formulas (2), (3), (4) and (5) to the differential DVHs exported from the treatment planning system. For the breast tumor radiobiological parameters were derived for the clinical data: α = 0.13 Gy^-1^ and α/β = 4.6 Gy [17].

The considered endpoints for heart toxicity were pericarditis and long term mortality. The NTCP for pericarditis was calculated using the LKB model with m = 0.13, n = 0.64, TD_50_ = 50.6 Gy and an α/β ratio of 2.5 Gy [21,22]. For long term mortality an α/β ratio of 3 Gy and the following parameters TD_50_ = 52.3 Gy, n = 1 and m = 0.28 were considered. This last value was found to give the best approximation to the Erikson breast dose effect curve [23] using the LKB model with TD_50_ and n fixed as in Gagliardi et al. [22,24]. The NTCP for LAD toxicity was calculated with the values n = 0.35; m = 0.1; TD_50_ = 48 Gy [25]. For lung toxicity we considered pneumonitis as endpoint and used TD_50_ = 30.8 Gy, m = 0.37 and n = 0.99 with an α/β ratio of 3Gy [26].

### Statistical analysis

The dosimetric data of PTV, contra-lateral breast, heart and ipsilateral lung and LAD, as well as the TCP and NTCP values were compared between the different breathing techniques.

Although the number of patients was very small a standard statistical assessment of the significance of the results was performed. Two tailed paired t-test was used to estimate the statistical significance of the differences between groups. A p-value less than 0.05 was considered statistically significant.

## Results

The standardized breath-hold procedure was easily understood by the patients and the training of the breathing pattern took a maximum of 30 minutes. By using eyeglasses the breath-hold technique was well accepted with a mean duration of 21 s (range: 15–48 s).

During the FB scans, the mean value over all patients of the vertical (antero-posterior) motion amplitude of the RPM box was 7 mm (range of 4 –11 mm). During DIBH the mean of the maximum amplitudes was 17 mm (range: 8–27 mm), i.e. a relative increase of 142.9% with respect to FB was found.

**Figure 1:**
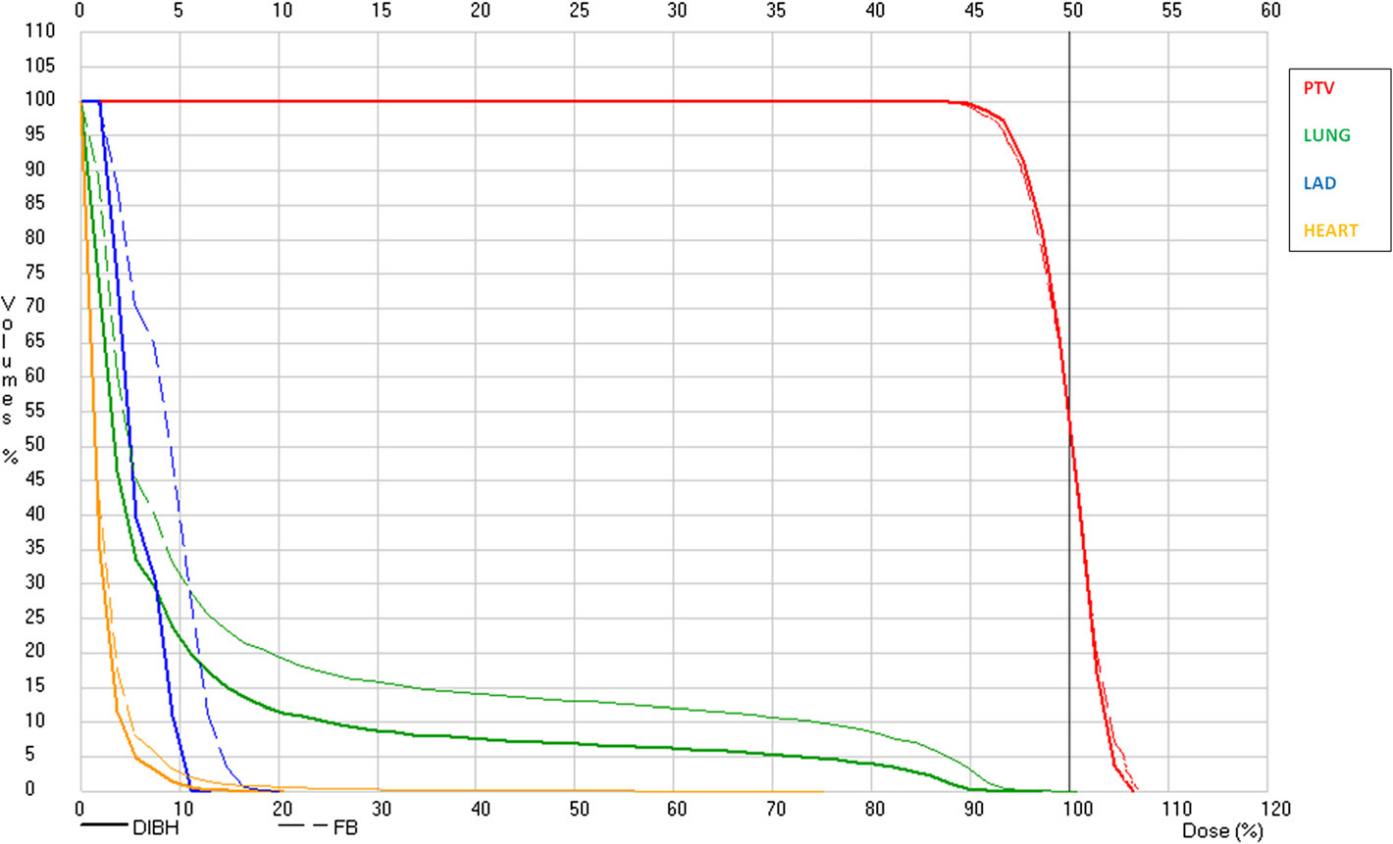
**Cumulative DVH showing how the dose to the critical organs is reduced between FB (thin dashed lines) and DIBH (thick continuous lines).** A standard schedule of 50 Gy/2 Gy fraction is considered.

A cumulative DVH representing the typical change between FB and DIBH in the dose distributions of the PTV and relevant normal tissues is shown in Figure 1.

### Pulmonary doses

CLD did not extend beyond 2.5 cm, regardless of whether the patient was in a FB or in a DIBH state.. No statistically significative difference in CLD values was found between DIBH and FB (p = 0.99).

A significant (p = 0.04) 28.7% increase in the patient averaged ILV was found in DIBH with repect to FB, however when the normalized ILV averaged over all patients was taken into account a 23.0% decrease was found, as shown in Table 1.

**Table 1:** Absolute lung volume, ILV and percentage normalized ILV in FB and DIBH

	**Absolute lung volume (cm**^**3**^**)**		**ILV (cm**^**3**^**)**		**Normalized ILV (%)**	
**Patient #**	**DIBH**	**FB**	**DIBH**	**FB**	**DIBH**	**FB**
1	1822.47	1428.66	81.10	67.29	4.45	4.71
2	2580.95	1313.33	97.56	43.34	3.78	3.30
3	2659.73	1539.35	199.48	180.72	7.50	11.74
4	1660.88	1165.16	71.75	59.19	4.32	5.08
5	2342.99	1483.92	75.21	71.97	3.21	4.85
6	1928.90	1068.35	192.89	122.54	10.00	11.47
7	2309.26	1301.86	177.12	118.99	7.67	9.14
8	2156.90	1209.99	64.06	81.19	2.97	6.71
All Pt Average	2182.76	1313.83	119.90	93.15	5.49	7.13

The mean (range) and p-values of IL mean dose (Dmean) and IL volumes receiving more than 10 Gy (V_10_) and 20 Gy (V_20_) are shown in Table 2 for FB and DIBH for both the conventional and the hypofractionated schedules.

**Table 2:** **Ipsilateral mean lung dose and lung volumes receiving more than 10 Gy (V**_**10**_**) and 20 Gy (V**_**20**_**)**

	**Conventional fractionation**			**Hypofractionation**		
	**DIBH**	**FB**	**p-value**	**DIBH**	**FB**	**p-value**
Dmean (Gy)	4.64	5.51	0.0505	3.15	3.75	0.0505
	(3.32 – 6.11)	(3.54 – 8.84)		(2.25 – 4.16)	(2.40 – 6.01)	
V_10_ (%)	9.08	11.54	0.0520	8.32	10.70	0.0405
	(5.52 – 15.44)	(6.46 – 19.46)		(4.93 – 14.22)	(5.79 – 17.92)	
V_20_ (%)	6.11	8.13	0.0398	5.71	7.65	0.0406
	(3.43 – 1.06)	(3.97 – 14.11)		(3.14 – 10.52)	(3.62 – 13.41)	

In the conventional fractionation the IL mean dose was reduced by 18.8% in DIBH. The mean values for V_10_ were 11.54% and 9.08% for FB and DIBH, respectively, which amounted to a 21.3% decrease in DIBH. In the hypofractionated schedule the IL mean dose was reduced by 16.0% in DIBH the mean values of V_10_ were 10.7% and 8.32%, respectively i.e. showed a 22.2% decrease in DIBH.

The V_20_ values were 8.13% and 6.11% for FB and DIBH, respectively, for the conventional schedule (24.8% decrease in DIBH). For hypofractionaction they were 7.65% and 5.71%, respectively (25.4% decrease in DIBH).

### Cardiac doses

The mean value over all patients of the minimum distance between the heart and the target volume was 1.43 cm (range 0.77-2.29 cm) for the FB CT datasets and 2.62 cm (range 2.12-3.25 cm) for the DIBH datasets. This 83.2% increase in DIBH was statistically significant (p = .0.0001).

In Table 3 the mean (range) and p-values of Dmean and Dmax of both heart and LAD are shown, for both conventional and hypofractioanted schedules and for both FB and DIBH. In addition the V_20_ and V_40_ for the heart are reported.

**Table 3:** The mean (range) and p-values for Dmean, Dmax of both heart and LAD

		**Conventional fractionation**			**Hypofractionation**		
**Organ**	**Parameter**	**DIBH**	**FB**	**p-value**	**DIBH**	**FB**	**p-value**
**Heart**	Dmax (Gy)^(*)^	5.00	29.19	0.0015	3.85	24.75	0.0025
		(2.00 – 10.00)	(5.00 – 52.00)		(1.00 – 8.00)	(3.00 – 46.00)	
	Dmean (Gy)	1.24	1.68	0.0106	0.84	1.14	0.0106
		(1.03 – 1.43)	(1.29 – 2.48)		(0.70 – 0.97)	(0.87 – 1.68)	
	V_20_^(**)^ (%)	0.00	0.39	0.1574	0.00	0.33	0.1644
		(0.00 -0.00)	(0.00 -1.61)		(0.00-0.00)	(0.00 – 1.40)	
	V_40_^(**)^ (%)	0.00	0.16	0.1719	0.00	0.07	0.1708
		(0.00 -0.00)	(0.00 – 0.70)		(0.00-0.00)	(0.00 -3.00)	
**LAD**	Dmax (Gy)^(*)^	4.25	19.62	0.0488	3.10	16.75	0.0479
		(2.00 – 11.00)	(3.00 – 52.00)		(1.00 – 8.00)	(2.00 – 46.00)	
	Dmean (Gy)	2.74	9.01	0.0914	1.86	6.12	0.9140
		(0.80 – 7.55)	(1.45 – 28.05)		(0.54 – 5.13)	(0.99 - 19.07)	

^(*)^EQD_2_ values using α/β =2.5 Gy for Pericardites in heart an for LAD.

^(**)^EQD_2_ values using α/β =3.0 Gy for long term Mortality.

As shown in the Table 3 the maximum doses to the heart and LAD and the mean dose to the heart were significantly lower in DIBH, (minimum 78.3% and 2.6% decrease with respect to FB, respectively) regardless of the schedule type.

In our series the maximum dose to LAD exceeded 20 Gy in 3/8 patients in FB, while it was lower than 20 Gy in all patients in DIBH.

### TCP and NTCP analysis

The TCP and NTCPs for lung and heart are reported in Table 4 as mean values with ranges. TCP values were increased in the hypo-fractionated schedule, as expected from the literature [17]. The NTCPs for Lung toxicity and long term cardiac mortality were at least 11.2% lower for DIBH with respect to FB, but the difference was statistically significant only for the long term cardiac mortality in the conventional fractionation. The NTCP for pericarditis and for LAD toxicity were 0% in all cases.

**Table 4:** TCP and NTCP for FB and DIBH

	**Conventional fractionation**			**Hypofractionation**		
**Parameter**	**DIBH**	**FB**	**p-value**	**DIBH**	**FB**	**p-value**
TCP (%)	96.40	96.30	0.3604	99.99	100.00	0.3506
	(92.5 – 98.23)	(94.33 – 97.36)		(99.97 – 100)	(100.00- 100.00)	
Heart NTCP (%) [pericarditis]	0.00	0.00	------	0.00	0.00	------
	(0.00 – 0.00)	(0.00 – 0.00)		(0.00 – 0.00)	(0.00 – 0.00)	
Heart NTCP (%) [long term mortality]	0.71	0.80	0.0385	0.72	0.87	0.0667
	(0.69 – 0.74)	(0.72 – 0.99)		(0.69 – 0.75)	(0.73 – 1.22)	
Lung NTCP (%) [pneumonitis]	6.58	11.48	0.2212	16.71	29.26	0.1618
	(0.23 – 13.18)	(0.77 – 33.54)		(8.19 – 29.43)	(9.57 – 97.70)	

## Discussions

The aim of this paper was to investigate clinical and dosimetric benefits of DIBH gating technique. The implementation of this practice allowed us to understand the factors influencing the correctness of this irradiation modality.

In particular, patient training is an important component of a clinical program that uses breath-hold respiratory gating for treatment [27]. It allows the patient to become familiar with the equipment and procedure, and provides an evaluation of the patient’s ability to perform reproducible breath-holds. In our experience the duration of the training session was reduced to 30 minutes. Lung inflation during inspiration increases the absolute lung volume but decreases the percentage irradiated lung volume (Table 1). Indeed, in 7 out of 8 patients the increase in ALV overcompensated the increase in ILV. Thus the mean lung dose should decrease, however the differences between DIBH and FB in our series showed only a trend (p-value = 0.05). In particular V_20_ was statistically significantly reduced in both the investigated schedules, while the reduction of V_10_ using DIBH was confirmed only in the hypofractionated schedule.

The published literature clearly indicates the need to reduce the irradiated heart volume as much as possible, even if there are no data from literature able to correlate a given risk of cardiac complication with some specific irradiated volume, such as LAD [25].

V_20_ and V_40_ for the heart were lower than 10% and 5%, respectively, which are the constraints for long term cardiac mortality [25,28].

The advantage of DIBH is to decrease the heart volume included in the irradiation fields, decreasing both the mean and the maximum dose of heart in a statistically significant way.

The difference in LAD maximum dose between DIBH and FB was statistically significant, while no statistically significant difference was found in the mean dose. Since the dose gradient is very steep on the internal side of the photon field, the increase of the distance between the target and the heart is very effective at decreasing the LAD maximum dose. On the other hand the lower doses which contribute to the mean dose are less affected by the distance increase. The maximum doses received by any part of the LAD should be lower than 20 Gy, according to Aznar et al. [25]. TCP calculation of both techniques revealed, as expected, a similar tumor control. When the NTCP models were applied, the difference observed for long term mortality was statistically significant only for the conventional fractionation. For the pericarditis endpoint, no differences were observed in both fractionation schedules. These results need to be confirmed because the small number of patients does not allow a statistic strong enough to state definitive conclusions. In addition the parameters of the NTCP/TCP models are generally derived using values from the literature which were derived using “static” or “averaged on respiratory cycle” CT images. Besides a careful follow up of the clinical outcome of these patients and the addition of more patients to the study, the investigation of lung density related parameters could further elucidate the dosimetric benefits of DIBH gating technique.

## Conclusion

In summary, radiation delivery in inspiration breath-hold conditions can reduce the dose delivery to the surrounding normal structures, in particular cardiac doses, due to the increase of the distance between target and heart as shown by our data and also confirmed by other authors [16]. As a result a consistent reduction in NTCP is achieved, with no loss in tumour control. Moreover our results suggest that DIBH, with proper patient selection and training, is a practical and achievable solution for minimizing respiratory-induced target motion during both simulation and treatment. On the negative side the use of gating techniques with breath-hold increases treatment room occupation due to a more complex set-up. Treatment time is also increased when multiple breath-holds and consequent breathing recovery intervals are needed to complete the irradiation of a beam. However this latter side effect could be compensated by decreasing the beam-on time with an increase in the dose rate.

## Consent

Written informed consent was obtained from the patient for the publication of this report and any accompanying images.

## Competing interests

All authors declare that they have no competing interests.

## Authors’ contributions

Conception and design: VB, EI, PP and LS. Target and OAR delineation in TC: CG and AMF. Collect data: AA and VB. Analysis and interpretation of the data: LS, AA and VB. Drafting of the manuscript: VB, EI, AA, VL, MD, AS, PP and LS. Final approval of the article: All authors read and approved the final manuscript.
